# The fields of HIV and disability: past, present and future

**DOI:** 10.1186/1758-2652-12-28

**Published:** 2009-11-09

**Authors:** Jill Hanass-Hancock, Stephanie A Nixon

**Affiliations:** 1 Health Economics and HIV/AIDS Research Division (HEARD), University of KwaZulu-Natal, Durban, South Africa; 2 Department of Physical Therapy, University of Toronto, Canada, and Research Associate, HEARD, University of KwaZulu-Natal, Durban, South Africa

## Abstract

This article provides an historic overview of the fields of disability and HIV. We describe this area of concern in terms of "fields" versus "a single field" because of the two related but distinct trends that have evolved over time. The first field involves people living with HIV and their experiences of disability, disablement and rehabilitation brought on by the disease and its treatments. The second involves people with disabilities and their experiences of vulnerability to and life with HIV. These two fields have evolved relatively independently over time. However, in the final section of this article, we argue that the divide between these fields is collapsing, and that this collapse is beginning to produce a new understanding about shared concerns, cross-field learning and the mutual benefits that might be realized from integrating policy and programmatic responses. We close by identifying directions that we expect these merging fields to take in the coming years.

## Introduction

This article provides an historic overview of the fields of disability and HIV. We describe this area of concern in terms of "fields" versus "a single field" because of the two related but distinct trends that have evolved over time.

**Figure 1:**
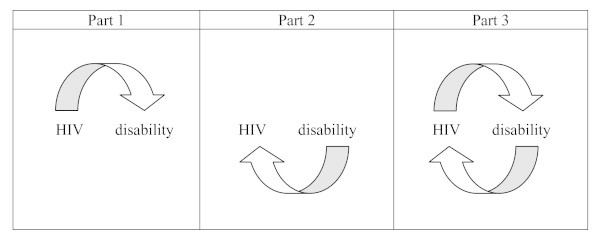
**The three dimensions of the HIV-disability field evolving over time**.

The first field involves people living with HIV and their experiences of disability, disablement and rehabilitation brought on by the disease and its treatments. The second involves people with disabilities and their experiences of vulnerability to and life with HIV. These two fields have evolved relatively independently over time. However, in the final section of this article, we argue that the divide between these fields is collapsing, and that this collapse is beginning to produce a new understanding about shared concerns, cross-field learning and the mutual benefits that might be realized from integrating policy and programmatic responses (see figure 1). The histories described in this article draw heavily on experiences in Canada, western Europe and southern Africa, and the particular advances that have taken place within these contexts.

### A note about definitions of disability

It is important to point out that in this article, the term, "disability", is used in different ways. Disability means different things to different groups of people, some of which are more politically charged than others and some of which have more positive or negative connotations.

In Part 1, we use the term, disability, largely as it has been described in the HIV literature. In this sense, it refers to the disabling effects of HIV, its secondary conditions and the side effects of medications. These disabling effects may be episodic and unpredictable, or permanent. However, another common understanding of the term is in reference to disability grants or benefits, which are typically a government subsidy (for example, in South Africa) for people unable to work because of a long-standing ailment or condition, which can include HIV.

In Part 2, we use the term, disability, largely as it has been used within the "disability community", which refers to the movement driven by people with auditory, visual, physical and intellectual impairments and their advocates. Although there is much debate in this community about theories of disability, the social model of disability is one leading approach that is often used to highlight the disabling role of society on individuals in contrast to more medicalized definitions.

The UN Convention of the Rights of Persons with Disabilities further states that: "Persons with disabilities include those who have long-term physical, mental, intellectual or sensory impairments which in interaction with various barriers may hinder their full and effective participation in society on an equal basis with others."[1] Whether this definition will be able to serve as a bridge between these two fields has yet to be seen. However, most important is to note that issues of language are important when discussing disability and have helped to shape the discourses in these two fields.

## Part 1: People living with HIV and experiences with disability

The story of HIV and disability mirrors the political history of HIV itself, both in terms of the (in)attention paid to the plight of people in poor versus wealthy countries until the recent past, and the delivery of HIV treatment in these two environments. The International AIDS Society's (IAS's) International AIDS Conferences, biannual global meetings on HIV and AIDS, provides a framework for telling this story.

### 1996-1998 - good news and bad news

The mood at the XI International AIDS Conference in Vancouver in 1996 was excited and optimistic. A new class of antiretrovirals (ARVs) called protease inhibitors had been discovered. When added to the two existing classes of ARVs, the result was the then-called "drug cocktail" (now called combination therapy, or highly active antiretroviral therapy, HAART), which appeared to be bringing people "back to life"[2]. It was good news indeed.

Two years later, at the XII International AIDS Conference in Geneva in 1998, the atmosphere was far more dismal. Not only had it been discovered that HAART produced a myriad of side effects (ranging from bothersome to fatal), but research was also showing that the positive effects of HAART were difficult to maintain over time due to drug resistance. As people living with HIV "failed on treatment" (a phrase which should be reversed to reflect "treatment failing people"), new regimens had to be introduced, with uncertainty about both the potential positive and adverse effects of the drugs.

As a result, in a few short years in the latter half of the 1990s, the experience of living with HIV for people who could access these new treatments had shifted from, typically, a fairly quickly progressing terminal illness to a life of hope combined with uncertainty. People were living longer, but with new experiences of episodic illness and disablement as a result of secondary effects of HIV (i.e., a broad range of HIV-related conditions that previously had not had time to surface), as well as the side effects of treatment [3].

Thus, it was the advent of HAART in developed countries that led to a response from governments, clinicians and, most significantly, the HIV community that sought to address this new experience of living with HIV.

### Rehabilitation in the context of HIV

From a clinical perspective, although medicine was best positioned to help contend with disease processes, it was the rehabilitation community (e.g., physical therapists, occupational therapists, speech-language pathologists and physiatrists) who brought expertise in dealing with the life-related consequences of the illnesses [4]. Furthermore, it was rehabilitation and disablement frameworks to which scholars and activists turned for insight into how to reconceptualize HIV beyond the level of disease. The World Health Organization's (WHO's) International Classification of Impairments, Disabilities and Handicaps (which was updated in 2001 and renamed the International Classification of Functioning, Disability and Health, or ICF) provided a framework that could highlight the challenges related to living with HIV at the level of the body structure or function (e.g., painful knee or congested lungs), the level of the individual (e.g., difficulty walking or getting dressed), and the level of involvement in life situations (e.g., difficulty with one's job or in parenting roles) [5,6]. This reframing provided the basis for both programming and policy advocacy.

For instance, the Canadian Working Group on HIV and Rehabilitation (CWGHR) was founded in 1998 by HIV activists, rehabilitation professionals, government policy makers and representatives from the insurance industry to examine and respond to the emerging needs of people living with HIV in this new context. Guided by the WHO framework, the organization's research and policy work has focused on such issues as work and employment, HIV education and mentorship for rehabilitation professionals, and the facilitation of a prevalence study to assess the level of disablement among populations of people living with HIV [7,8].

In 2005, Worthington *et al *advanced a conceptualization of rehabilitation in the context of HIV that was informed by these efforts and based directly on the ICF [9]. This HIV Rehabilitation Conceptual Framework heightens understanding of rehabilitation domains, services and issues in the context of HIV. Using the ICF, the framework outlines the multiple life domains affected by HIV and associated treatments, provides a working definition of rehabilitation in the context of HIV, and highlights the expanded role that health providers and services have in the rehabilitation of people living with HIV, including their role in enhancing their labour forces and overall social participation. Although this framework is being taken up in certain environments in wealthy countries, it has had only limited application in resource-limited settings [10].

### A new concept: episodic disability

**Figure 2:**
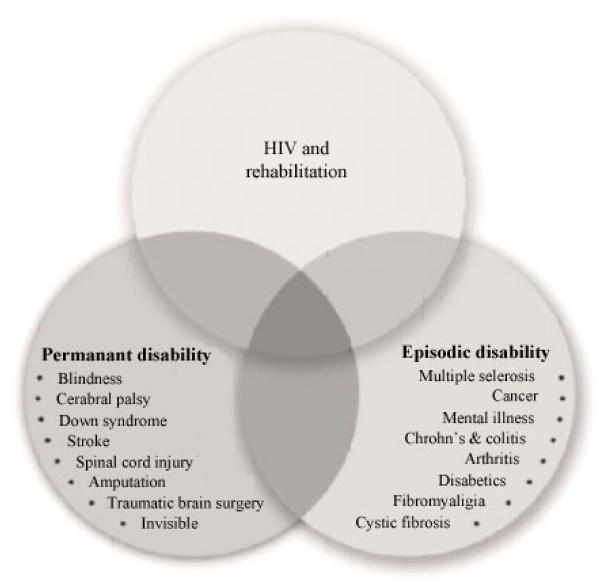
**Conceptual model of cross-disability issues developed by the Canadian Working Group on HIV and Rehabilitation**.

Along with advocacy efforts geared solely to HIV, the CGWHR also brought together like-minded individuals and organizations from outside of the HIV world that were facing similar concerns. An early outcome of this "cross-disability" initiative was the creation of a model (see figure 2), which helped identify areas of shared concern across the groups and sparked the notion of "episodic disability". The model uses medical diagnoses as the basis for illustrating the intersection of issues related to: HIV; "permanent" or static disabilities; and "episodic" disabilities, which refer to experiences of disablement that are unpredictable and intermittent in nature.

While there are issues shared across each sphere, a particular affinity was discovered between HIV and other lifelong, episodic conditions. This recognition of the unpredictable nature of living with HIV has proven to be a crucial milestone in the Canadian context for advancing policy advocacy. For instance, this realization led to collaborative cross-disability efforts between HIV and other advocacy groups, including: development of the Statement of Common Agenda on Episodic Disabilities; joint meetings with government representatives and decision makers involved in income support and employment programmes; and a national multi-sectoral summit on episodic disabilities in 2006 [11].

Episodic disability in the context of HIV has been further understood through research by O'Brien *et al *that explored how adults living with HIV conceptualize disability [12]. Participants perceived disability as a term that suggested permanency in contrast to their experiences of episodic illness. However, participants were willing to adopt the description of disability in order to access crucial social services. Participants explained that the term, "disability", on its own did not capture their experiences. Rather, the term, "episodic disability", emerged as a more accurate framing of the variable health-related consequences experienced by adults living with HIV [13].

This research on people living with HIV also led to development of the Episodic Disability Framework, which describes disability as episodic and multi-dimensional in nature, characterized by unpredictable periods of wellness and illness. The framework consists of three main components:

a) Dimensions of disability (symptoms and impairments, difficulties carrying out day-to-day activities, challenges to social inclusion, and uncertainty) that may fluctuate on a daily basis and over the course of living with HIV;

b) Contextual extrinsic factors (social support and stigma) and intrinsic factors (living strategies and personal attributes) that may exacerbate or alleviate disability; and

c) Triggers that initiate momentous or major episodes of disability [12].

This idea has been the basis for practical applications, such as the identification of policy models to promote more flexible income support and employment programmes to enable people with episodic disabilities to work when their health permits without losing their income support or health benefits if they get sick again, or to work part-time on an ongoing basis combined with partial disability income support. A second application has been the development of educational curricula for employers, human resource professionals and vocational counsellors regarding accommodation of people with episodic disabilities in the workplace. Finally, this framing has led to the development of new models of care for people with episodic disabilities whose health status and health care needs tend to fluctuate.

As people on HAART live longer lives, the long-term impacts of HIV and its treatments, in combination with aging itself, may include increased prevalence of co-morbidities, such as arthritis, fractures from osteoporosis, diabetes, some forms of cancer, and depression or other mental illnesses [14], all of which may also be episodic in nature and impact. As such, people living with HIV may experience several episodic conditions concurrently, all with different fluctuations in their functioning and health. Thus, the corresponding need for rehabilitation is being seen to expand in order to prevent or manage such disabling impacts and maintain or promote improved quality of life.

This framing of disability stands in contrast to other leading conceptualizations. First, its fundamental concern with cycles of health and illness exists in opposition to the social model of disability, which locates society as the site of the problem, as opposed to the body. Second, the definition of disability in the UN Convention on the Rights of Persons with Disabilities includes the requirement of the impairment being "long term" [1]. The fit between this definition and that of episodic disability has yet to be theorized.

### 2000 - HIV becomes political

Returning to the timeline traced by the International AIDS Conferences, the 2000 meeting was held in Durban, South Africa. This was the first time that a resource-limited country had played host. It was at this conference that science was met head on by politics. The focus on HIV treatments that had dominated the previous two meetings was displaced by a new emphasis on a profound global disparity with respect to attention, resources and research for HIV. More than 90% of people living with HIV resided in poor settings, yet access to HIV treatments in those regions was available to only a handful of elites. As such, activists argued that it was unjust for attention at a global meeting to centre on the science behind treatment without simultaneously addressing the vast political chasm of concern between rich and poor countries.

At that point, the cost of treatment was so prohibitively high that it precluded serious contemplation of universal coverage in poor countries. However, 2001 saw a dramatic drop in the price of treatment due in large part to the competitive market created by low-cost generic versions of the HIV drugs by Indian pharmaceutical companies that were not limited by World Trade Organization patent regulations.

In 2002, the XIV International AIDS Conference in Barcelona, Spain, witnessed a shift that reflected newfound commitment to redressing the disparity in access between rich and poor countries with respect to HIV treatment, as well as other aspects of HIV prevention, care and support. The World Health Organization announced its bold "3 by 5" campaign, which promised to have three million people on HIV treatment by 2005. Two years later, the theme of the 2004 conference, held in Bangkok, Thailand, was *Access For All*. The question of whether or not universal access was possible had shifted to how best to achieve this goal. Although unforgivably late and tragically slow, the move to deliver HIV treatment to all in need was in motion.

With the arrival of treatment in poor countries, so the issue of rehabilitation in the context of HIV surfaced in these parts of the world. Some issues have mirrored concerns raised in environments like Canada, such as the challenge of living with an unpredictable and episodic illness when one's financial safety net is tied to health and employment status [15]. However, new issues have emerged in research, such as paediatric rehabilitation interventions: the number of children living with HIV in high-prevalence countries is significantly higher than in wealthy countries. It is also important to note that issues of disability and rehabilitation are not limited to people on treatment. Although the advent of treatment has prompted action in this field, there are concerns and opportunities for intervention for people who either cannot access or tolerate the drug regimens.

Thus, the field of disability and rehabilitation in the context of HIV has gone global, and one may expect that interest in these issues will expand as treatment becomes more of a reality in poor countries.

### A field becomes established

The field of disability, disablement and rehabilitation in the context of HIV has come a long way in just over a decade. Along with the new geographic reach, there are now bodies of research on a range of topics, including: assessment of disablement among people living with HIV [10], HIV and exercise [16], HIV and rehabilitation best practices [17], preparedness of rehabilitation professionals to treat people living with HIV [18], and barriers and facilitators to labour force participation.

Furthermore, in 2007, the Canadian Working Group on HIV and Rehabilitation undertook a scoping review to identify key research priorities in HIV and rehabilitation to advance policy and practice for people living with HIV in Canada. Among the research priorities that emerged were: further exploring the prevalence and impact of disability among people living with HIV; better understanding the episodic nature of disability as it fluctuates over time; and exploring the impact of episodic disability on one's overall health [12].

It is important to note that the degree of engagement on issues of rehabilitation and disability in the context of HIV described in Canada do not necessarily reflect a general trend worldwide. There is work to be done in more broadly advancing the issue in other regions. However, it is also noteworthy that the Canadian response has largely been located in a health paradigm, which has resulted in limited collaboration with disability organizations.

More progressive intersections between the HIV and disability communities can be found in other countries, such as Australia, where the Disability Discrimination Act was passed in 1992. The Act's definition of disability included "the presence in the body of organisms capable of causing disease or illness", thus including people living with HIV [19]. There have also been notable activists, such as John Campbell of the United Kingdom, who was a person living with HIV and a disability activist. As chair of the British Council of Disabled People and founder of an organization for people who are hearing impaired, Campbell was visionary in recognizing challenges shared by people with disabilities and people living with HIV [20].

Another conceptual leap, however, involves people with pre-existing disabilities and their vulnerability to HIV; it is the history of this movement that forms Part 2 of this article.

## Part 2: People with disabilities and experiences with HIV

The response to HIV and AIDS can largely be characterized as the identification and targeting of key populations that are believed to be at increased risk of exposure to HIV. These populations of people are now well known, including, depending on region and epidemic, migrant workers, sex workers, men who have sex with men, injecting drug users, and indigenous people. Even in generalized epidemics, as seen in southern Africa, prevention and care efforts have focused, in part, on those perceived to be most at risk, such as youth in resource-poor settings. People with disabilities have for a long time been excluded from any discussion of key populations at increased risk for HIV. However, the logic underpinning this exclusion has been flawed and in their recent policy brief UNAIDS acknowledges people with disabilities as a key population at higher risk of exposure to HIV [21].

### Myths about people with disabilities are debunked

First, the assumption that people with disabilities comprise only a small minority is incorrect. The World Health Organization estimates that one in 10 people in the world lives with some sort of disability [22]. However, this 10% is not distributed evenly around the world; as is the pattern with many challenges, the prevalence of disabilities in resource-limited settings outweighs that of more wealthy countries. As such, one can assume that more than 10% of the population has a disability in places like southern Africa, where HIV prevalence is also at its highest.

Second, the abiding assumption that people with disabilities are at little or no risk for HIV was disproved in the Global Survey on HIV/AIDS and Disability, a seminal World Bank study conducted by Nora Groce in 2004 [23]. Data collected from organizations working with people with disabilities in 57 countries across four continents concluded that almost all known risk factors for HIV and AIDS are increased for people with disabilities [24]. The eight areas of vulnerability identified in the survey continue to be proven through additional empirical research. The following section highlights evidence supporting our understanding of why people with disabilities are at increased risk for HIV.

**1. Poverty**: People with disabilities are often the poorest members of their communities, and the World Bank estimates that persons with disabilities may account for 20% of the poorest citizens in the world [23-25].

**2. Lack of education**: People with disabilities are typically excluded from school because they are not considered in need of education, are assumed to be a distraction in class, or are believed to be incapable of learning [23,26]. Even when in school, children with disabilities are less likely to receive science and health education and more likely to be excused from sex education courses [24,27-29].

**3. Lack of HIV and "safer sex" information resources**: There is a pervasive misperception that people with disabilities are asexual. Although adolescents with disabilities are generally more socially isolated, they have been shown to be as sexually experienced as their able-bodied peers [23,30,31]. Youth with disabilities have also reported double the rates of ever having had a sexually transmitted disease or being pregnant than their able-bodied counterparts [32]. A systematic review has also revealed that people with disabilities in Africa are as sexually active as the general population, yet sexuality is still not addressed [33]. Reproductive health awareness-raising programmes are known to frequently exclude people with disabilities [34-37]. Individuals with disabilities are rarely the targets of HIV interventions designed specifically to address their particular prevention needs [38] and are less likely to have access to condoms or other prevention methods [26].

**4. Elevated risk for violence and rape, and lack of legal protection**: Abuse among women with disabilities ranges from double to quadruple the rate found among women in general [23,24,39-42]. Approximately 80% to 90% of persons with disabilities are victims of some type of abuse at some point in their lives [38]. Adult women with a disability are more likely than non-disabled females to be physically or sexually assaulted by their partners and women with disabilities are more likely to be subjected to serious violence [32]. However, legal protection is still lacking [43-47].

**5. Substance abuse**: Drug abuse among select groups of people with disabilities is reported to be significantly higher than the general population [26,28]. Substance use is associated with elevated sexual risk taking [23,48] and may also lead to sharing injecting equipment, resulting in increased vulnerability to HIV.

**6. Vulnerability of disabled orphans**: Children with disabilities who are orphaned have been found to be particularly vulnerable as they are losing a parent and are less likely to receive the same care and support as their non-disabled orphaned peers [24].

**7. Precarious access to affordable health care**: Health care providers have been reported to routinely deny people with disabilities access to HIV testing and HIV and AIDS care [24]. Lower priority is often placed on individuals with disabilities when scarce HIV medications and services are being rationed [23,26]. Furthermore, people with disabilities face barriers to accessing any form of health care services (e.g., because clinics are missing ramps and Braille or sign interpreters), which can result in other sexually transmitted infections being undiagnosed, further increasing risk of HIV infection.

**8. Stigma**: Stigma has been associated with HIV, as well as with disability. People with disabilities who become HIV positive may become doubly stigmatised [24]. A further layer of discrimination may also be experienced by people who are not heterosexual [49].

It is now understood that people with disabilities are at least as much, if not more, at risk of HIV infection than the general public. However, studies evaluating HIV prevalence rates among people with disabilities are only now beginning to emerge. The first prevalence studies were conducted with deaf populations and demonstrated that deaf people are at least as likely [50,51], if not twice as likely [52], to become HIV positive as non-deaf controls. Although the samples were relatively small, the results provide important early verification of arguments advanced in the Global Survey on HIV/AIDS and Disability.

With these myths debunked, the field of disability and HIV is now emerging. Before looking further ahead, however, we will glance backwards to reflect on the evolution of this arm of the HIV and disability story.

### 2001 - Evidence emerging from Africa

Whereas stories of the earliest activism on HIV began with the voices of people living with HIV in America and Europe, so this arm of the story begins with people who have disabilities, and professionals working in the field of disability. Unlike the history of HIV, however, this story has a strong link to Africa, where it embraced development concerns from the start.

It was disabled people's organizations and disabled service organisations in Africa that raised concerns about HIV. These are organisations driven largely by people with disabilities, their parents, and other caregivers that provide services and advocate with and for people with disabilities. They enjoy a long history in both rich and poor countries and have an infrastructure that spans the local, national, regional and global levels.

Within poor countries, disabled people's organizations and disability services typically subscribe to a community-based rehabilitation model. Community-based rehabilitation is a community development strategy, which sits in contrast to institutional-based rehabilitation approaches (e.g., rehabilitation services based in hospital settings). It promotes not only technical rehabilitation solutions, but also the equalization of opportunities and social integration of all people with disabilities [53,54]. Furthermore, community-based rehabilitation (CBR) provides services that reach into rural areas that are otherwise underserved by formal health care. This model of care focuses on working with local people and training them to use indigenous materials to enable basic rehabilitative interventions and care. It was within this network of care that HIV was identified as a threat in southern Africa [54-56].

In the early 2000s, some disabled people's organizations in Africa identified that their caregivers and CBR workers were becoming infected and affected by HIV. Disability programmes, like Comprehensive Community-Based Rehabilitation Tanzania, started offering HIV services (e.g., voluntary counselling and testing, and home-based care) in parallel to their disability programming in response to this emerging need within their communities [54]. It was soon realized, however, that HIV services were needed by people with disabilities themselves [24,55].

The first mention in the literature of people with disabilities in Africa at risk for HIV arrived in 2000 when Osowole and Oladimeji [57] described an evaluation of a peer-based HIV prevention intervention conducted in two Nigerian schools that enrolled both deaf and hearing students. The intervention was shown to increase knowledge about HIV but to have no effect on perceptions of personal susceptibility to HIV. Comparison to hearing population was not reported.

In 2002, the international development agency, Save the Children, commissioned a study to explore approaches to engaging youth in the response to HIV [58]. The project involved action research interventions in two communities that had previously been overlooked in HIV prevention responses: youth in a rural community in South Africa, and youth in a school for the blind. Thus, although there were important pockets of awareness prior to 2004, the Global Survey on HIV/AIDS and Disability filled a significant research gap and catalyzed a collection of advocacy and research activities [23,24,59].

### 2004 - Recognition from the north: a symposium in Germany

In 2004, a landmark symposium on disability and HIV was coordinated in Germany by a network called People with Disability in the One World [55]. This symposium brought together academics, service providers and disabled people's organizations from Europe, North America and Africa to discuss the vulnerability of people with disabilities to HIV. The symposium included the presentation of some of the first research in the area, including preliminary results from the Global Survey on HIV/AIDS and Disability.

This academic information was complemented by insights from service providers and disabled people's organizations. For example, a representative from the international non-governmental organization, Handicap International, described its programmes that address the vulnerability of people with disabilities, which had been initiated in 2000 [60]. One of these programmes, based in Kenya, sought to make voluntary counselling and testing (VCT) and other prevention interventions accessible for blind people. A second programme, operating in France, focused on people with intellectual disabilities and involved discussion groups of people with disabilities, their caretakers and professional counsellors to discuss sexual and reproductive health. The discussions aimed to open up space to address issues of intimacy among people with intellectual impairments and, thus, decrease the stigma surrounding issues of sexuality for these people with disabilities [55].

The director of Comprehensive Community-Based Rehabilitation Tanzania described the experience in Tanzania, where HIV was increasingly affecting the disabled population [61]. He explained that the question had shifted from "if" to "how" rehabilitation programmes should get involved in HIV, but lamented the fact that few programmes had adapted to meet these evolving needs.

Also at the symposium was Disabled People South Africa, a disabled people's organization that emerged in 1984 as a direct result of the double discrimination facing black people with disabilities under the apartheid regime in South Africa [62]. This organization was represented by prominent activists, Emily Ntuli and Andrew Dube, who described the disability and HIV situation in their country as desperate [63,64]. HIV services were described as inaccessible for people with disabilities. Furthermore, stigmatization, sexual abuse and exploitation were flagged as factors driving the epidemic within the disabled population. Dube called for strategies in three fundamental areas:[63]

1. Implementing interventions to build awareness around HIV and sex education for youth with disabilities;

2. Expanding research on disability and HIV, particularly in the context of sexual and reproductive health and gender-based violence; and

3. Promoting safer sexual practices among people with disabilities in a manner that is consistent with the best available research (noting that the legitimacy of mainstream HIV research was being questioned by the South African government at the time).

### 2006 - National disability advocacy at the epicentre of the HIV epidemic: South Africa

In 2006, the South African Department of Health began developing a new strategic plan for HIV and AIDS, and called upon all sectors to provide input. The disability sector in South Africa was well organized. The highlight of this advocacy effort was the forming of the South African Disability Alliance (SADA) formerly known as the South African Federal Council on Disability (SAFCD). Through them the disability sector was represented at the South African National AIDS Council. The sector convened to develop input for the draft South African National Strategic Plan 2007-2011. Through these efforts, people with disabilities became recognized as a vulnerable group and sector within the new National Strategic Plan [49,65,66]. As a result, South Africa is recognized as one of the few countries in Africa that has comprehensively incorporated concern for people with disabilities into its HIV strategy [21].

### 2007 - Regional disability advocacy: the Africa Campaign on Disability and HIV/AIDS

At the regional level, 2007 saw the birth of the Africa Campaign on Disability and HIV/AIDS [67,68]. This movement was spearheaded through the joint leadership of Handicap International and the Secretariat of the African Decade of Persons with Disabilities (1999-2009). The goal of the African Decade of Persons with Disabilities is the full participation, equality and empowerment of people with disabilities in Africa; one of its five programmes focuses on HIV [69].

The Africa Campaign on Disability and HIV/AIDS was launched in January 2007 at a meeting that brought together disabled people's organizations and service providers from African countries. The objectives of the campaign were to promote: a coordinated response involving persons with disabilities in African countries to achieve inclusive national HIV and AIDS policies and programmes; and equal access for persons with disabilities in Africa to information and services on HIV and AIDS [70].

At its second meeting, held in Uganda in 2008, the campaign launched the Kampala Declaration, which calls for "all governments to include disability in its diversity as a cross-cutting issue in all poverty reduction strategies" [71]. The Kampala Declaration has since been used as an education and advocacy tool with African governments, and was at the centre of the campaign's activities at the third meeting, which was held on the margins of the International Conference on STIs and HIV/AIDS (ICASA) in Dakar, Senegal, in December 2008. At this meeting, the campaign lobbied for the needs of people with disabilities by posing disability-related questions to conference presenters and through facilitating special sessions that focused on disability and HIV.

### The rise of HIV research on and with people with disabilities

In the five years since the results of the Global Survey on HIV/AIDS and Disability were released, research on people with disabilities and their experiences with HIV has grown exponentially. Returning to the lens of the International AIDS Conferences, at the 2004 meeting in Bangkok, Prince Ngongo Bahati won a Young Researcher Award for his study on voluntary counselling and testing for people who are deaf. His work had been conducted in collaboration with Liverpool VCT Kenya, an organization working with and for deaf people, which has developed a leading model of VCT peer-counselling for people with disabilities [50,72].

At the XVI International AIDS Conference in Toronto in 2006, the Canadian Working Group on HIV and Rehabilitation and the International Centre for Disability and Rehabilitation held two sessions on HIV and disabilities as a way to promote dialogue on these issues. Another event at the conference was entitled "Deaf People and HIV/AIDS: Time to Recognize the Problem". Interest in this area was starting to grow.

**Figure 3:**
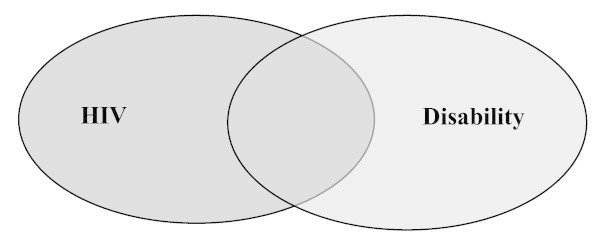
**Integration of the fields of HIV and disability**.

By the time the XVII International AIDS Conference was held in Mexico City in 2008, disability held a significant place in the programme. A session in the formal programme, entitled "Beyond Barriers: Disabilities and AIDS", plus other research papers, were devoted to issues facing people with disabilities (see Appendix 1). There were also four disability-related satellite sessions led by AIDS-Free World, Disabled People International, Voluntary Service Overseas, the Catholic Organisation for Relief and Development Aid (Cordaid), and the Inter-American Institute on Disability and Inclusive Development (IIDI). Building on this momentum, the 2008 ICASA meeting, convened later that year, included two sessions focusing on disability and HIV (see figure 3). A further milestone was the 3^rd ^LAC Technical Meeting on STDs, HIV/AIDS and Disability, held just prior to the conference. The event was co-organized by IIDI, the World Bank, the National Council to Prevent Discrimination (Mexico), the Central American Social Integration System, the National Program of STI and AIDS (Brazil), and the Pan-American Health Organization.

This dramatic rise in interest at the International AIDS Conference in 2008 prompted the creation of an Internet-based network on disability and HIV. This network is now a vibrant communication exchange tool with more than 160 researchers, activists and other stakeholders sharing information about research, advocacy developments, policies, meetings and publication opportunities [73]. Other networks, like the International Disability and Development Consortium (IDDC), which advocates internationally for the inclusion of disability, have also taken the issue of disability and HIV on board [74].

### The emergence of accessible HIV resources and services

Issues of accessibility are beginning to be addressed. For example, the International AIDS conferences in 2006 and 2008 were criticized for the lack of accessibility for people with disabilities at the conference sites. However, a team has been established to plan for enhancing accessibility and inclusion of people with disabilities at the XVIII International AIDS Conference to be held in Vienna, Austria, in 2010.

Other initiatives to enhance accessibility of HIV services and educational materials for people with disability are being piloted. For example, recognizing the need for VCT counsellors trained in working with people with disabilities, the African Union of the Blind has produced a "Train the Trainer" manual that targets service providers to better understand the needs of people who are blind [75,76]. To address the challenge of sexuality, intimacy and HIV with people with intellectual disabilities, teaching materials have been developed with particular focus on poor countries [77,78].

In 2004, the Liverpool VCT, Care and Treatment disability programme in Kenya was launched to provide HIV services to deaf people [50,79]. It also produced a sign language manual, entitled "Signs for Sexuality and Reproductive Health", and is now training other staff in Kenyan sign language. The programme, run entirely by deaf counsellors and administrative staff, is the only one of its kind in Africa. Notably, many sign languages are missing signs for sexual- or HIV-related issues (e.g., Mexican sign language); however, it may be possible to borrow ideas from the Kenyan model.

Another challenge involves engagement with the legal system for people with disabilities who are involved in sexual abuse and/or gender-based violence, especially in resource-limited countries. To respond to this need, the Cape Mental Health Organisation in Cape Town, South Africa, developed a comprehensive counselling intervention to support people with intellectual disabilities during trials for rape [44,80].

Other disability organizations that have become involved in HIV issues include: CBR Education and Training for Empowerment (CREATE) in South Africa, which is involved in VCT programmes; Christian Blind Mission, which funds work in the field; Enablement in the Netherlands, which offers courses on HIV and disability; and the Brazil Universidade of Mackenzie initiative, which has developed a video addressing sexuality and human rights for youth with intellectual disabilities. Although coverage falls far short of demand, the advent of these programmatic responses indicates that issues of HIV and disability are beginning to receive recognition and, surely, we will soon see the first best-practice collection arriving in the field.

### The rise of policies to support people with disabilities and HIV

Several significant policy efforts related to this field have taken place or are underway. The United Nations Convention on the Rights of People with Disabilities, which came into force in 2008, has generated much interest in issues of disability [1]. In 2009, the Joint United Nations Programme on HIV/AIDS (UNAIDS) released the Disability and HIV Policy Brief [21].

Also in 2009, the Government of Canada convened an international policy dialogue exploring the intersection of HIV and disability [81]. Country-level policies to support people with disabilities are at different stage of development. In Africa, for example, some countries have included disability within their national strategic frameworks or plans, although they are of different depths (e.g., Uganda, Tanzania, Zambia, Swaziland, Lesotho and South Africa). Regional bodies, like the Southern African Development Community Parliamentary Forum is preparing an HIV/AIDS model law to act as a framework for countries in the region, although it is yet to be seen how well it addresses issues of disability.

## Part 3: Increased integration of the fields of HIV and disability

Although the two fields may have unique histories, there is now evidence that they are becoming increasingly integrated (see figure 3). First, we are seeing HIV organizations taking up the concerns of people with disabilities. For example, a leading voice of people with disabilities at the International AIDS Conference in Mexico City in 2008 was AIDS-Free World, the non-governmental organization spearheaded by Stephen Lewis, former UN Special Envoy for HIV/AIDS in Africa. Second, we are seeing disability organizations taking up the concerns of people living with HIV. Third, there are examples of HIV organizations and disability organizations meeting in the middle ground to address shared concerns.

For example, the Disability and HIV/AIDS Trust, based in Botswana, operates as an umbrella organization for the southern African region to bring together disabled people's organizations and AIDS service organizations. Another illustration is the recent International Policy Dialogue on HIV/AIDS and Disability, hosted by the Government of Canada, which engaged representatives from each field to explore synergies. Looking to the future, there are various directions that we anticipate these fields to take, based on current trajectories.

### Human rights as a unifying advocacy tool

The United Nations Convention on the Rights of Persons with Disabilities is a major advance for people with disabilities and their advocates. Not only is this spotlight bringing attention to disability issues, but the focus on a rights-based approach to these concerns is crucial. This development has attracted the attention of HIV communities and will likely serve as a vehicle for further integrating the two movements.

We are likely to see the HIV community looking to the convention for opportunities to realize rights in a new way, and the disability community looking to the HIV community for additional lessons learned through successful human rights advocacy. With the increasing feminization of the HIV pandemic, plus the disproportionate burden that women face with respect to physical and mental disabilities, gender and the link with sexual and reproductive rights will likely emerge as a focus of concern within the HIV and disability realm.

### Wider recognition of the ICF framework

There is the potential for a constructive tension to be generated by the collision of the different conceptual orientations that have underpinned the evolution of the two fields. For example, rehabilitation and disability in the context of HIV draws on a medical model, which is concerned with diagnosis and disease-level issues. The movement has broadened to engage issues at the societal level. However, the link to health remains firmly entrenched.

Conversely, the disability movement has a tradition based on the social model of disability and reactions to it. A human rights framework provides an alternative to each of the approaches and will serve to advance the fields, as discussed above. However, it is also likely that a conceptual middle ground will be found in the International Classification of Functioning, Disability and Health (ICF) model, which incorporates many dimensions of both of these frameworks and may offer a common language across the fields.

### Focus on service delivery

The field of rehabilitation in the context of HIV was spurred by the arrival of treatment more than a decade ago in wealthy countries. With treatment now rolling out in resource-limited countries, a similar phenomenon is likely to arise whereby people with HIV start living longer lives, but with a range of activity limitations and participation restrictions. Thus, the need for disability and rehabilitation services will grow.

However, this growth in demand for services will occur in regions where health and social service systems are already fragile and where many people with disabilities are already underserved, putting extraordinary pressure on already stretched systems. One response will see the parallel systems of home-based care and community-based rehabilitation being sought to help fill the gaps. The models underpinning home-based care, which is a common model of care for HIV and AIDS, and community-based rehabilitation, a dominant approach to disability, derive from similar philosophies (see table 1).

**Table 1:** Contrasting community-based rehabilitation and home-based care

	Community-based rehabilitation	Home-based care
**Definition**	A strategy within community development for the rehabilitation, equalization of opportunities, and social integration of all people with disabilities.	Any form of care given to ill people in their homes, including physical, psychological, palliative and spiritual activities.
		
	Implemented through the combined efforts of disabled people themselves, their families and communities, and the appropriate health, education, vocational and social services [82].	The goal is to provide hope through high-quality and appropriate care that helps ill people and families maintain their independence and achieve the best possible quality of life [8].

**Target group**	People with disabilities	People with HIV and other chronic or disabling conditions

**Setting**	Resource-limited settings	Resource-limited settings

**Type of care**	Rehabilitation and care	Care and rehabilitation

As these similarities come to be understood across the two fields, opportunities for cross-learning can be realized, particularly from community-based rehabilitation because of its long history (e.g., using community-based rehabilitation handbooks for guidance in using local resources, and learning lessons about financing home-based care based on the decades of experience in community-based rehabilitation programmes). We can also expect to see the development of best-practice guidelines for HIV and disability care and support.

### Mainstreaming of disability concerns in HIV

The concerns of people with disabilities will increasingly be taken seriously and mainstreamed into HIV research, policy and programmes. This engagement will grow because of leadership at the grassroots level by disabled people's organizations and service delivery organizations, as well as internationally by such champions as AIDS-Free World. We are also likely to see the emergence of charismatic champions, who are people with disabilities and also live with HIV.

Despite momentum in this direction, a key obstacle to progress will be discrimination from people living with HIV toward the disability community, and vice versa. This cross-discrimination has its origin in people living with HIV not wanting to be seen as disabled, and people with disabilities not wanting to be seen as sick [21]. Difficult questions regarding mainstreaming HIV as a disability may arise for countries that have disability grant and benefit systems that were previously closed to people living with HIV. Similarly, interpreting HIV as a disability has implications for disability statistics, and it may be feared that the political power of HIV will steal attention and support from other disability issues.

Returning to the framework of the International AIDS Conferences, however, there are a series of positive firsts that are likely to unfold at the XVIII International AIDS Conference in Vienna in 2010. First, the conference will see new, high-quality empirical research on disability and HIV. Second, disability will not only have its own session, but will be mainstreamed across the programme. For example, we may see a presentation on disability and sexual exploitation nested within a session on sexual abuse and HIV. Third, disabled people's organizations will have a significant presence in the community village, where much advocacy and information exchange takes place. Finally, the Vienna gathering will be the most accessible and inclusive of all International AIDS Conferences to date.

## Conclusion

This article has attempted to chart of the fields of HIV and disability over time, in both their parallel paths and, finally, in their more integrated form. This history has drawn heavily on experiences within southern Africa, Europe and Canada, with acknowledgement that there must be other aspects to the histories that have not been represented. It is our hope that this seminal effort will spur others around the world to add to this history, both by sharing experiences to date and by taking up concern with HIV and disability issues as we move forward together.

## Competing interests

The authors declare that they have no competing interests.

## Authors' contributions

SN and JHH have contributed equally to this article. In particular, SN wrote the first draft of Part 1, and JHH wrote the first draft of Part 2. The third section developed jointly by the two authors.

## Appendix 1

### Examples of research on disability and HIV presented at major HIV conferences in 2008

• Bisol showed that risk of HIV is higher for deaf people in Brazil who have lower levels of HIV knowledge, less formal school education, and higher rates of sexual abuse. (WEAD0204. Mexico 2008).

• Touko reported prevalence rates that demonstrated deaf people in Cameroon are as likely to get infected with HIV as their non-disabled peers. (WEAD0205, Mexico 2008)

• Monaghan reported on a US study that found VCT uptake among the deaf was lower and that HIV infection was higher than the national average. (Mexico 2008)

• Henderson presented results from the Steadman Group Study on HIV and AIDS Knowledge, Attitude, Practice and Accessibility with a deaf population in Kenya. (74, Dakar 2008)

• Vidal reported on neurological disabilities in AIDS patients. (SAT session, Mexico 2008)

• Guimaraes described risk behaviours among patients with chronic mental illness in a national multicentre study in Brazil. (WEDA0202, Mexico 2008)

• Hanass-Hancock reported on a study examining the interweaving patterns of disability, gender and HIV and AIDS, which highlighted the problem of sexual abuse and exploitation among people with disabilities in South Africa. (WEAD0203, Mexico 2008)

• Hanass-Hancock presented a systematic literature review on HIV and disability in Africa. (76, Dakar 2008)
